# Irritable bowel syndrome increases the risk of chronic obstructive pulmonary disease: A retrospective cohort study

**DOI:** 10.1038/s41598-020-66707-1

**Published:** 2020-06-19

**Authors:** Hsiang-Chun Lai, Hung-Jen Lin, Yi-Wei Kao, Kai-Hsun Wang, Jen-Wei Chou, Ben-Chang Shia, Sheng-Teng Huang

**Affiliations:** 1 Department of Chinese Medicine, China Medical University Hospital, Taichung, Taiwan; 2 School of Chinese Medicine, China Medical University, Taichung, Taiwan; 3 Graduate Institute of Business Administration, Fu Jen Catholic University, Taipei, Taiwan; 4 Research Center of Big Data, College of management, Taipei Medical University, Taipei, Taiwan; 5 Division of Gastroenterology and Hepatology, Department of Internal Medicine, China Medical University Hospital, Taichung, Taiwan; 6 Executive Master Program of Business Administration in Biotechnology, College of management, Taipei Medical University, Taipei, Taiwan; 7 Cancer Research Center for Traditional Chinese Medicine, Department of Medical Research, China Medical University Hospital, Taichung, Taiwan; 8 Chinese Medicine Research Center, China Medical University, Taichung, Taiwan; 9 Research Center for Chinese Herbal Medicine, China Medical University, Taichung, Taiwan; 10 An-Nan Hospital, China Medical University, Tainan, Taiwan

**Keywords:** Gastroenterology, Medical research

## Abstract

Both inflammation and infection are associated with the development of irritable bowel syndrome (IBS) and chronic obstructive pulmonary disease (COPD). The purpose of this study is to further elucidate the association between IBS and COPD through a retrospective cohort study. We enrolled IBS patients diagnosed between 2000 and 2011 with follow-up for at least one year. The non-IBS patients as comparison group were selected with 1:3 matching by propensity score. Statistical analysis was utilized to assess the differences in characteristic distribution, and to compare the cumulative incidence of COPD between the IBS and non-IBS cohorts. We selected 14,021 IBS patients and 42,068 non-IBS patients for comparison. The IBS patients exhibited a significant risk to develop COPD compared with non-IBS patients. Additionally, the cumulative incidence rate of COPD in the IBS cohort increased significantly during the follow-up period of more than ten years, compared to the non-IBS cohort, based on the Kaplan-Meier analysis. The risk of COPD was also significantly decreased in those patients with more than eighteen IBS-related clinical visits. This retrospective cohort study demonstrates the significantly increased risk of COPD in patients with IBS. Therefore, early inspection and prevention of COPD is essential for patients with IBS.

## Introduction

Irritable bowel syndrome (IBS) is a common functional gastrointestinal (GI) disease, more commonly affecting women than men. The global prevalence of IBS reported is 11%; with the highest reported rate in South America (21.0%), and the lowest in Southeast Asia (7.0%)^1^. Based on the newest Rome IV system, IBS was diagnosed as recurrent abdominal pain at least 1 day/week on average during the prior 3-month period, with patients exhibiting symptoms including a change of abdominal pain pattern after defecation, and a change in the frequency of stool or the form of stool, based on the Bristol stool form scale^2^. These symptoms were generally believed to be related to IBS alone, with no association with any other organic diseases. Meanwhile, Paudel *et al*. has reported that 27% of IBS patients were associated with organic lesions, according to the Rome IV criteria, the most prevalent being nonspecific colitis, ileal erosions, polyps, hemorrhoids, and diverticula^3^. The pathophysiology of IBS includes intestinal permeability, gut immune dysfunction, gut bacteria flora overgrowth/alteration, gut motility, visceral sensation, brain-gut axis dysregulation, and changes to psychological status^4^. Comorbidities of IBS are often related to GI disorders, including functional dyspepsia and gastroesophageal reflux disease (GERD); however, comorbidities may be related to extra-GI disorders, such as asthma, fibromyalgia, chronic pelvic pain, interstitial cystitis, migraine, chronic fatigue syndrome, depression, and anxiety^5–8^.

Chronic obstructive pulmonary disease (COPD) is an obstructive lung disease characterized by shortness of breath and productive cough^9^. The global prevalence rate of COPD is reported to range from 7% to 12%, affecting males more than females, and resulting in significant social and economic burden^10^. Comorbidities of COPD include pulmonary hypertension, hypertension, congestive heart failure, coronary heart disease, metabolic syndrome, obstructive sleep apnea syndrome, asthma, and GERD^11,12^. Several factors have been reported to trigger COPD, including smoking, genetic determinants, cancer growth, infection, environmental stimuli, and progressive immunological disorders^13^.

Both inflammation and infection models have been utilized in the explorations of IBS and COPD pathophysiology, including investigations into the changes to microbiota, which may play an important role in IBS patients^14^. Although several studies have suggested a higher prevalence of GI disorders among COPD patients, especially GERD^15–17^, less research has explored the correlation between IBS and COPD^18^. We propose that there exists an association between IBS and COPD due to their similar pathophysiological pathways. Herein is a population-based retrospective cohort study of the Taiwan National Health Insurance Research Database (NHIRD) to investigate the association between IBS and COPD.

## Results

### Demographic characteristics

There were a total of 14,021 subjects included in the IBS cohort, and 42,068 subjects in the non-IBS cohort, as shown in Table 1. The data distribution between the two cohorts was basically similar in terms of sex, residential area, salary, age distribution, smoking-related diseases/conditions, comorbidities, and drug use. Females exhibited a higher incidence of IBS in comparison with men (men: women = 45.3: 54.7). People living in the northern region of Taiwan had a higher percentage (46.4%) of IBS compared to inhabitants of the other regions. It is noteworthy that those patients with higher stated income had lower incidence of IBS (25.7%). The IBS patients aged from 40–59 had the highest incidence (43.3%) compared to those less than 40 years of age, and older than 60 years of age (28.1%, 28.6% respectively). The subjects visited the tobacco quit clinic in both IBS and non-IBS cohorts were 215 (1.5%) and 552 (1.3%) respectively with no statistical significance. In the comparison of comorbidities between the two cohorts, no statistical significance was noted, except for hypertension (p value = 0.001), and GERD (p value < 0.0001). In addition, no statistical significance was noted in COPD medications between the IBS and non-IBS cohorts.

**Table 1:** Demographic Characteristics and comorbidities of patients newly diagnosed with IBS and non-IBS in Taiwan.

Variables	IBS				*p*-value
	Yes (N = 14,021)		No (N = 42,068)		
	n	%	n	%	
Sex					0.911
Male	6354	45.3	19089	45.4	
Female	7667	54.7	22979	54.6	
Area					0.987
Northern	6506	46.4	19551	46.5	
Central	3512	25.0	10569	25.1	
Southern	3748	26.7	11194	26.6	
Eastern	255	1.8	754	1.8	
Salary					0.597
≤NT$15,840	4956	35.3	14936	35.5	
NT$15,841-25,000	5456	38.9	16484	39.2	
≥NT$25,001	3609	25.7	10648	25.3	
Age (years)					0.799
18~29	1660	11.8	5023	11.9	
30~39	2292	16.3	6784	16.1	
40~49	3154	22.5	9349	22.2	
50~59	2913	20.8	8648	20.6	
60~69	2045	14.6	6263	14.9	
>69	1957	14.0	6001	14.3	
Smoking-related diseases/conditions					0.056
Yes	215	1.5	552	1.3	
No	13806	98.5	41516	98.7	
Comorbidity					
hypertension	3167	22.6	10085	24	0.001
coronary heart disease	986	7.0	2949	7.0	0.944
ischemic stroke	139	1.0	407	1.0	0.842
hemorrhagic stroke	45	0.3	177	0.4	0.121
diabetes mellitus	1481	10.6	4588	10.9	0.264
renal insufficiency	194	1.4	577	1.4	0.949
depression	417	3.0	1177	2.8	0.290
asthma	329	2.3	1046	2.5	0.370
GERD	174	1.2	310	0.7	<0.001
anxiety	947	6.8	2870	6.8	0.796
osteoporosis	485	3.5	1361	3.2	0.208
peptic ulcer	720	5.1	2260	5.4	0.288
Medication					
steroids	180	1.3	538	1.3	>0.999
anti-cholinergics	27	0.2	70	0.1	0.600
beta-2 agonist	155	1.1	487	1.2	0.650

IBS = irritable bowel syndrome, COPD = chronic obstructive pulmonary disease, GERD = gastroesophageal reflux disease;

Chi-square test was used to test categorical variables.

### The cause-specific hazard ratio and cumulative incidence of COPD

**Figure 1:**
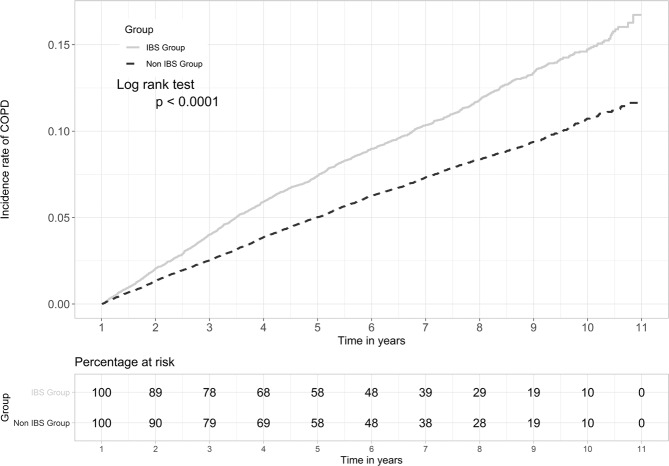
Cumulative incidence of COPD in the patients with IBS and non-IBS by Kaplan-Meier method analysis. The statistical analyses were performed using R software Version 3.4.3 (http://www.r-project.org), with level α = 0.05 to indicate statistical significance.

According to the Cox proportional hazards model, the factors affecting COPD risk between the IBS and non-IBS patients included gender, age, salary, residential area, smoking-related diseases/conditions, comorbidities, and COPD medications (Table 2). The results showed that the IBS cohort presented an increased risk of developing COPD compared to the non-IBS cohort (adjusted HR = 1.512, 95% CI = 1.414–1.617, p value < 0.0001). Of note, during the study period, the cumulative incidence of COPD was significantly higher among IBS patients as compared with non-IBS patients (Fig. 1). In addition, an increased risk of COPD incidence in both cohorts was noted in the male population (aHR = 1.610, 95% CI = 1.508–1.720, p value < 0.0001), and the eastern region inhabitants (aHR = 1.243, 95% CI = 1.012–1.526, p value = 0.0377); whereas a decreased risk of COPD incidence was noted in the population with higher reported income (aHR = 0.834, 95% CI = 0.749–0.928, p value = 0.0008). Meanwhile, age seems to be an important factor, with an increased risk of developing COPD trending parallel with increased age. Positive smoking-related diseases/conditions posed a higher risk of COPD (HR = 1.695, 95% CI = 1.292–2.224, p value = 0.0001), while didn’t reach a statistical significance in adjusted HR (aHR = 1.190, 95% CI = 0.905–1.546, p value = 0.2123) (Table 2). Those IBS patients with comorbidities including hypertension, coronary heart disease, ischemic stroke, hemorrhagic stroke, diabetes mellitus, renal insufficiency, depression, asthma, peptic ulcer, anxiety, sleep apnea, and osteoporosis exhibit a higher risk of developing COPD compared to the non-IBS group. However, only coronary heart disease, diabetes mellitus, depression, asthma, peptic ulcer, anxiety, sleep apnea, and osteoporosis comorbidities reached statistical significance (Table 2). This result indicates that there are many diseases associated with IBS patients, possible enhancing the risk of COPD presentation. The effects of IBS on COPD occurrence depending on age, gender, and the presence of comorbidities were demonstrated in Table 3. Among age evaluation, the patients with IBS had higher risk of COPD than those without IBS in all group (aHR = 1.31–1.87, p < 0.001). The effect of IBS on COPD incidence was significantly higher in both men (aHR = 1.50) and women (aHR = 1.55) as well as participants both with (aHR = 1.76) and without any comorbidities (aHR = 1.39). Based on a stratification analysis of the 2, 4, 6, 8, and 10-year follow-up periods, we found that within all of the follow-up period, the IBS cohort demonstrated a significantly increased risk of COPD compared to the non-IBS cohort, as shown in Fig. 1.

**Table 2:** Cox model with hazard ratios and 95% confidence intervals of risk associated with COPD and comorbidities in patients with IBS compared to those without IBS.

Variables	Univariate		Multivariate	
	Crude HR (95%CI)	*P*-value	aHR (95%CI)^†^	*P*-value
Group				
Non-IBS	1	reference	1	reference
IBS	1.462 (1.367, 1.564)	<0.0001	1.512 (1.414, 1.617)	<0.0001
Sex				
Female	1	reference	1	reference
Male	1.480 (1.389, 1.577)	<0.0001	1.610 (1.508, 1.720)	<0.0001
Age				
18~29	1	reference	1	reference
30~39	1.652 (1.281, 2.131)	0.0001	1.629 (1.261, 2.105)	0.0002
40~49	2.852 (2.265, 3.591)	<0.0001	2.777 (2.200, 3.505)	<0.0001
50~59	5.405 (4.322, 6.759)	<0.0001	5.097 (4.062, 6.394)	<0.0001
60~69	9.480 (7.601, 11.824)	<0.0001	8.395 (6.704, 10.513)	<0.0001
>69	18.241 (14.675, 22.673)	<0.0001	15.022 (12.019, 18.776)	<0.0001
Area				
Northern	1	reference	1	reference
Central	1.123 (1.038, 1.215)	0.0038	1.020 (0.941, 1.107)	0.6275
Southern	1.160 (1.075, 1.251)	0.0001	1.024 (0.947, 1.106)	0.5551
Eastern	1.545 (1.261, 1.892)	<0.0001	1.243 (1.012, 1.526)	0.0377
salary				
≤NT$15,840	1	reference	1	reference
NT$15,841-25,000	0.882 (0.824, 0.944)	0.0003	1.056 (0.982, 1.136)	0.1404
≥NT$25,001	0.487 (0.442, 0.536)	<0.0001	0.834 (0.749, 0.928)	0.0008
Smoking-related diseases/conditions				
No	1	reference	1	reference
Yes	1.695 (1.292, 2.224)	0.0001	1.190 (0.905, 1.546)	0.2123
Comorbidities				
hypertension	2.431 (2.279, 2.593)	<0.0001	1.057 (0.982, 1.138)	0.1366
coronary heart disease	2.532 (2.314, 2.771)	<0.0001	1.191 (1.082, 1.310)	0.0003
ischemic stroke	2.996 (2.372, 3.784)	<0.0001	1.253 (0.989, 1.589)	0.0615
hemorrhagic stroke	2.673 (1.916, 3.728)	<0.0001	1.396 (0.998, 1.952)	0.0516
diabetes mellitus	1.894 (1.740, 2.062)	<0.0001	1.112 (1.016, 1.216)	0.0213
renal insufficiency	2.219 (1.810, 2.720)	<0.0001	1.131 (0.921, 1.389)	0.2385
depression	1.441 (1.220, 1.702)	<0.0001	1.352 (1.143, 1.598)	0.0004
asthma	2.788 (2.438, 3.188)	<0.0001	2.491 (2.177, 2.850)	<0.0001
GERD	1.570 (1.067, 2.310)	0.0221	1.074 (0.729, 1.582)	0.7172
anxiety	1.616 (1.453, 1.796)	<0.0001	1.155 (1.036, 1.287)	0.0093
sleep apnea	1.844 (1.047, 3.251)	0.0342	1.775 (1.006, 3.135)	0.0478
osteoporosis	2.025 (1.783, 2.300)	<0.0001	1.234 (1.082, 1.407)	0.0017
peptic ulcer	1.676 (1.493, 1.881)	<0.0001	1.164 (1.034, 1.310)	0.0122

Crude HR = relative hazard ratio, aHR = Adjusted hazard ratio, CI = confidence interval; IBS = irritable bowel syndrome, COPD = chronic obstructive pulmonary disease, GERD = gastroesophageal reflux disease.

^†^Model was adjusted for age, sex, and comorbidities.

**Table 3:** Incidence of COPD by age, sex and comorbidity and Cox model measured hazards ratio for patients with IBS compared those without IBS.

Variables	Irritable bowel syndrome						Crude HR (95% CI)	Adjusted HR^†^ (95% CI)
	No			Yes				
	Event	PY	IR^#^	Event	PY	IR^#^		
Age, years								
<=49	449	130574.55	3.44	283	44288.76	6.39	1.85(1.60, 2.15)***	1.87(1.61, 2.17)***
50-64	737	68117.56	10.82	407	22531.11	18.06	1.67(1.48, 1.89)***	1.68(1.49, 1.90)***
65+	1408	48534.21	29.01	573	15570.33	36.80	1.27(1.16, 1.40)***	1.31(1.18, 1.44)***
Sex								
Female	1173	135804.23	8.64	575	45573.91	12.62	1.46(1.32, 1.61)***	1.50(1.35, 1.65)***
Male	1421	111422.09	12.75	688	36816.29	18.69	1.47(1.34, 1.61)***	1.55(1.41, 1.69)***
Comorbidity^§^								
No	849	143727.02	5.91	475	47446.79	10.01	1.69(1.51, 1.90)***	1.76(1.57, 1.97)***
Yes	1745	103499.29	16.86	788	34943.41	22.55	1.34(1.23, 1.46)***	1.39(1.28, 1.51)***

Crude HR = relative hazard ratio, aHR = Adjusted hazard ratio, CI = confidence interval; IBS = irritable bowel syndrome, COPD = chronic obstructive pulmonary disease, IR = incidence rate, PY = person-years,

^†^Model was adjusted for age, sex, and comorbidities

# incidence rate, per 1000 person-years

§ Individuals with any comorbidity of hypertension, coronary heart disease, ischemic stroke, hemorrhagic stroke, diabetes mellitus, renal insufficiency, depression, asthma, gastroesophageal reflux disease, anxiety, sleep apnea, osteoporosis and peptic ulcer were classified into the comorbidity group.

***p < 0.001.

### The hazard ratios of COPD associated with COPD medication and frequency of outpatient service in IBS patients

Recent COPD treatments are designed to slow disease progression. Thus, we further analyzed the association of hazard ratios with COPD regimens, including steroids, anti-cholinergics and beta-2 agonists between the IBS and non-IBS cohorts. Our results found that a higher incidence rate of COPD generally demonstrated significant differences between the IBS and non-IBS cohorts with steroids and anti-cholinergics taken for less than 28 days (p value < 0.0001), but not for COPD medications taken for more than 28 days (Table 4). This indicates that patients with IBS had a higher possibility of developing COPD, and subsequently request COPD treatment. In addition, the regression model demonstrated that the aHR of COPD was higher in the IBS groups recording less than 18 times outpatient visits (aHR = 1.355–1.621, p < 0.05), reaching a statistical significance, rather than the IBS group recording more than 18 outpatient visits, (aHR = 1.077, 95% CI = 0.905–1.281, p = 0.4046) as compared to the non-IBS cohort (Table 5). This indicates that receiving treatment for IBS might decrease the incidence rate of COPD.

**Table 4:** Cox model with hazard ratios and 95% confidence intervals of COPD risk with medication treatment and covariates in patients with IBS compared to those without IBS.

Medication (days)	Univariate		Multivariate	
	Crude HR (95%CI)	*P*-value	aHR (95%CI) ^†^	*P*-value
Steroids				
less than 28 days				
non IBS	1	reference	1	reference
IBS	1.461 (1.365, 1.563)	<0.0001	1.514 (1.415, 1.620)	<0.0001
28 to 84 days				
non IBS	1	reference	1	reference
IBS	2.653 (1.117, 6.302)	0.0271	2.337 (0.926, 5.894)	0.0722
more than 84 days				
non IBS	1	reference	1	reference
I BS	0.318 (0.040, 2.545)	0.2804	0.122 (0.008, 1.912)	0.1339
Anti-cholinergics				
less than 28 days				
non IBS	1	reference	1	reference
IBS	1.464 (1.368, 1.566)	<0.0001	1.514 (1.415, 1.62)	<0.0001
28 to 84 days				
non IBS	1	reference	1	reference
IBS	0.580 (0.171, 1.97)	0.3825	0.181 (0.020, 1.677)	0.1325
more than 84 days				
non IBS	1	reference	1	reference
IBS	1.412 (0.56, 3.563)	0.4647	0.028 (0.001, 0.680)	0.0281
β2 agonist				
less than 28 days				
non IBS	1	reference	1	reference
IBS	1.459 (1.363, 1.562)	<0.0001	1.514 (1.414, 1.621)	<0.0001
28 to 84 days				
non IBS	1	reference	1	reference
IBS	1.569 (0.927, 2.656)	0.0934	1.170 (0.632, 2.165)	0.6175
more than 84 days				
non IBS	1	reference	1	reference
IBS	1.858 (0.926, 3.729)	0.0815	1.432 (0.619, 3.316)	0.4015

Crude HR = relative hazard ratio, aHR = Adjusted hazard ratio, CI = confidence interval, IBS = irritable bowel syndrome, COPD = chronic obstructive pulmonary disease

^†^Model was adjusted for age, sex, and comorbidities.

**Table 5:** Cox model with hazard ratios and 95% confidence intervals of COPD risk with frequency of outpatient visits for IBS.

	Events	Crude HR (95% CI)		aHR (95% CI)^†^	
Non-IBS	2594	1 (Reference)		1 (Reference)	
times of IBS OPD visits					
>0 & <=6	689	1.436 (1.320, 1.561)	<0.0001	1.621 (1.490, 1.763)	<0.0001
>6 & <=12	335	1.655 (1.477, 1.854)	<0.0001	1.618 (1.444, 1.814)	<0.0001
>12 & <=18	105	1.408 (1.158, 1.711)	0.0006	1.355 (1.115, 1.647)	0.0023
>18	134	1.252 (1.053, 1.490)	0.0111	1.077 (0.905, 1.281)	0.4046

Crude HR = relative hazard ratio, aHR = Adjusted hazard ratio, CI = confidence interval, IBS = irritable bowel syndrome, COPD = chronic obstructive pulmonary disease.

^†^Model was adjusted for age, sex, and comorbidities.

## Discussion

Although previous studies have demonstrated that patients with COPD have an increased risk of IBS^19,20^, this is the first study using a population-based database to show that IBS potentially increases incidence rates of COPD. Our findings suggest that IBS patients had a significantly higher risk of developing COPD (aHR = 1.512) after adjusting for sex, age, and major comorbidities.

**Figure 2:**
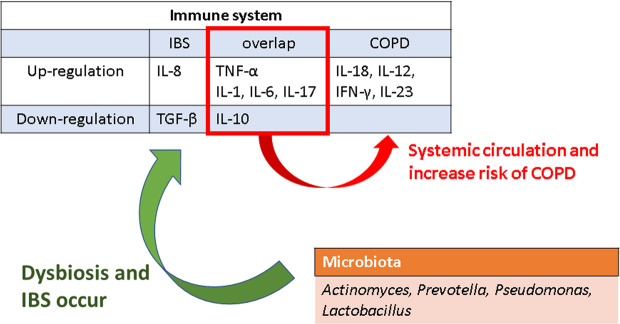
Possible mechanism and interaction between IBS and COPD.

Previous studies had mentioned the increased risk of inflammatory bowel disease (IBD) in COPD patients and the relationship of IBD and IBS. Labaraca *et al*. reported association between IBD and COPD and suggested potential mechanisms including increased pro-inflammatory cytokines, autoimmune antibodies, angiogenesis factors, and microbiome dysfunction, which leading to endothelial barrier dysfunction^21^. On the other hand, IBS and IBD shared many pathological mechanisms and overlapping symptoms. Spiller *et al*. demonstrated the shared mechanisms between IBS and IBD such as sustained submucosal inflammation, immune activation, dysbiosis in the gut and endothelial barrier dysfunction^22^. Other factors including stress, visceral hypersensitivity and the gut–brain axis were mentioned. Thus, we suppose that there would be some link between IBS and COPD. The immune system potentially plays a key role in the association between IBS and COPD. IBS patients have exhibited increased levels of TNF-α, IL-1, IL-6, IL-8, IL-17, and decreased levels of TGF-β, IL-10^23–25^. Furthermore, COPD results from sustained inflammation of the airways leading to the destruction of lung tissue and impairment of lung function. The inflammatory process is related to T cells, neutrophils, and macrophages triggered by cytokines. Recent studies have reported that COPD patients also had the effect to upregulate IL-1, IL-18, IL-6, IL-12, TNF-α, IFN-γ, IL-23, IL-17 and downregulate IL-10^26–28^. As such, there exist overlapping cytokine levels, including IL-10, TNF-α, IL-1, IL-6, and IL-17 between IBS and COPD. Furthermore, studies have demonstrated the role of infection associated with disturbances of the microbiota in patients with IBS^29^. Such exposure to pathogens interferes with the gut barrier and increases gut permeability. However, inflammatory signals not only affect the gut but also circulate throughout the body causing visceral hyperalgesia associated with the brain-gut model of IBS pathophysiology^30^. The composition of lung microbiota in COPD patients has been shown to differ from that of healthy controls^31,32^. The immuno-stimulating properties of bacteria in the airway microbiota are very similar to gut microbiota^14^. While species like *Actinomyces, Prevotella, Pseudomonas* and *Lactobacillus* have been detected in both lungs and gastrointestinal tract^14,31^. Based on these findings, we suppose that dysregulation of the immune system, partially attributed to disturbed microbiota activity, could potentially explain the increased risk of COPD in patients with IBS. (Fig. 2).

Previous studies have pointed out that IBS affects females more than males^33,34^. In a meta-analysis study, the prevalence of IBS in women was higher in comparison with men (14.0% vs 8.9%; OR = 1.67)^1^. In the present study, we included IBS patients with 1:3 propensity score matching, with women comprising more than men (M:F = 45.3: 54.7), which is consistent with the IBS sex ratio previously reported. On the other hand, COPD affects men more than women, with a reported OR of 1.55 to 1.7^35–38^, a trend which is further confirmed in this study (aHR = 1.610). We also note a steady increase of COPD diagnoses parallel with increasing age^10^. Pothirat *et al*. have reported that the general prevalence of COPD is higher in rural areas compared to urban areas (6.8% vs 3.7%)^39^. This study found similar result that in Taiwan, the prevalence of COPD is the highest in the eastern, a rural areas compared to urban areas and reached a statistical significance to the northern area, a densely populated cities. Of note, the prevalence rate of comorbidities including COPD, coronary heart disease, diabetes mellitus, peptic ulcer, asthma, depression, anxiety, sleep apnea, and osteoporosis were significantly higher in patients with IBS than controls, confirming previous reports that IBS patients with the abovementioned comorbidities had a higher risk of developing COPD than those without comorbidities^11,12^.

Treatments for COPD include beta-2 agonists, anti-cholinergics, steroids (inhaled or oral), theophylline, and phosphodiesterase type 4 inhibitor inhibitors. In addition, IBS patients treated with beta-2 agonists for less than 28 days had an increased incidence of developing COPD, with statistical significance. As reported, adrenergic stimulation triggers visceral hypersensitivity in IBS patients and rat model^40–42^; the mechanism of which is related to the polymorphism of adrenergic activation and elevation of transient receptor potential cation channel subfamily V member 1 (TRPV-1) sensitization^42–44^. Meanwhile, anticholinergic agents, which inhibit muscarinic receptors and cause smooth muscle relaxation^45^, may serve as effective antispasmodics and play an important role in control of abdominal pain in IBS patients^46^. Inhaled anticholinergic agents, such as ipratropium, oxitropium, and tiotropium also increase luminal diameter by smooth muscle relaxation and decreased submucosal gland mucin secretion in COPD patients^47^. We noted a decreased risk of COPD in IBS patients using anticholinergic agents for 28 to 84 days (aHR = 0.181, 95% CI = 0.020-1.677, p value = 0.1325) and for more than 84 days (aHR = 0.028, 95% CI = 0.001-0.680, p value = 0.0281) compared to the cohort receiving no drug treatment. The previously reported common physiological benefits of anticholinergic agents for treatment of both IBS and COPD are compatible with the findings of this study, although require further investigation^45^. We found that IBS patients with steroid treatment of more than 84 days had decreased incidence of COPD (aHR = 0.122) with no significance. Steroids affect metabolism, blood pressure regulation, balance of mood, and anti-inflammation. Corticosteroids also coordinate with beta agonists by up-regulation of β2-adrenoreceptors and modulate the immune system by regulation of T cells^48^, with a protective effect against development of COPD on patients using steroids for more than 84 days.

A higher rate of medical visits of IBS patients correspond with increased HR of COPD, indicating the severity and relapse rates of IBS patients; however, this consequence is decreased in patients recording medical visits of more than eighteen times. This suggests that the decreased incidence rate of COPD may be attributed to the active treatment of IBS. Importantly, IBS patients indeed exhibit an increased cumulative incidence rate of COPD, indicating significant association between these two diseases. However, the pathophysiology and specific interactions related to inflammation, infection, microbiota activity, and corresponding pathways between IBS and COPD remain unclear and require further investigation.

This is the first longitudinal population-based cohort study to evaluate the association between IBS and COPD. Due to the national health insurance (NHI) system covering 99.6% of the Taiwanese population as of 2017, this study has a large sample size yielding sufficient power for subgroup analysis, and enhanced potential to arrive at more conclusive results with adjustment for sex, age, and major comorbidities. NHIRD analysis is also inexpensive, with a very low loss of follow-up rate; thus, many studies using the available data are published annually. However, there remain several limitations and flaws to take into consideration. First, we used the ICD-9-CM algorithm to define diseases cited by clinical physicians. The precision of diagnoses was monitored by the Taiwan Bureau of National Health Insurance (an official insurance authority). The subjects were selected according to coding after a single inpatient admission, or three outpatient clinical visits to increase the validity and accuracy of the diagnoses. Second, the NHIRD does not offer information on potential confounding factors, including occupation, tobacco exposure, body mass index, physical activity, environmental/chemical exposure, or family history. We have analyzed the salary factor to discuss potential socioeconomic influence, which is consistent with other studies^39^. According to the Health Promotion Administration Report, tobacco usage was steady decrease in Taiwan recently. In 2017, 28.7% of adults above the age of 18 years used tobacco products in Taiwan general population^49^. However, there were no estimated data in IBS patients. Thus, we have adjusted the smoking factor for smoking-related diseases/conditions including personal history of tobacco use, tobacco use disorder, nonspecific abnormal results of pulmonary function studies, and other diseases of the lung (ICD 9: V15.82, 305.1, 794.2, 518) that are often diagnosed in the quit smoking clinic. Although underestimated smoking prevalence in our included patients, there was no statistical significance between two cohorts accordingly. Third, more detailed clinical evaluations, such as medical research council dyspnea scale, COPD assessment test, pulmonary function tests, serum laboratory data, or imaging results, are unavailable on the NHIRD. Thus, we cannot evaluate the severity of IBS in the subsequent risk of COPD, nor the COPD severity.

In conclusion, this retrospective cohort study indicates the significantly increased risk of developing COPD in patients with IBS. Instead of a causal relationship, both IBS and COPD may share a similar pathophysiological process associated with inflammation, infection, and microbiota interaction. However, further basic research is required to bridge these two diseases. Due to the increased COPD risk identified herein, it is critical for health care systems globally to enhance programs of early prevention and examination for COPD in patients with IBS.

## Materials and Methods

### Data sources

The data was retrieved from the (NHIRD), established by the National Health Research Institute (NHRI). The NHIRD is a population-based claims database covering over 99% of the Taiwanese population. The database is comprised of the claims data of over 23 million NHI enrollees during the years 2000 to 2011. All information, including age, sex, economic status, residence, diagnoses, dates of outpatient visits/admissions, and drug use have been verified. International Classification of Diseases, Ninth Revision, Clinical Modification (ICD-9-CM) codes is applied to classify diagnostic diseases.

**Figure 3:**
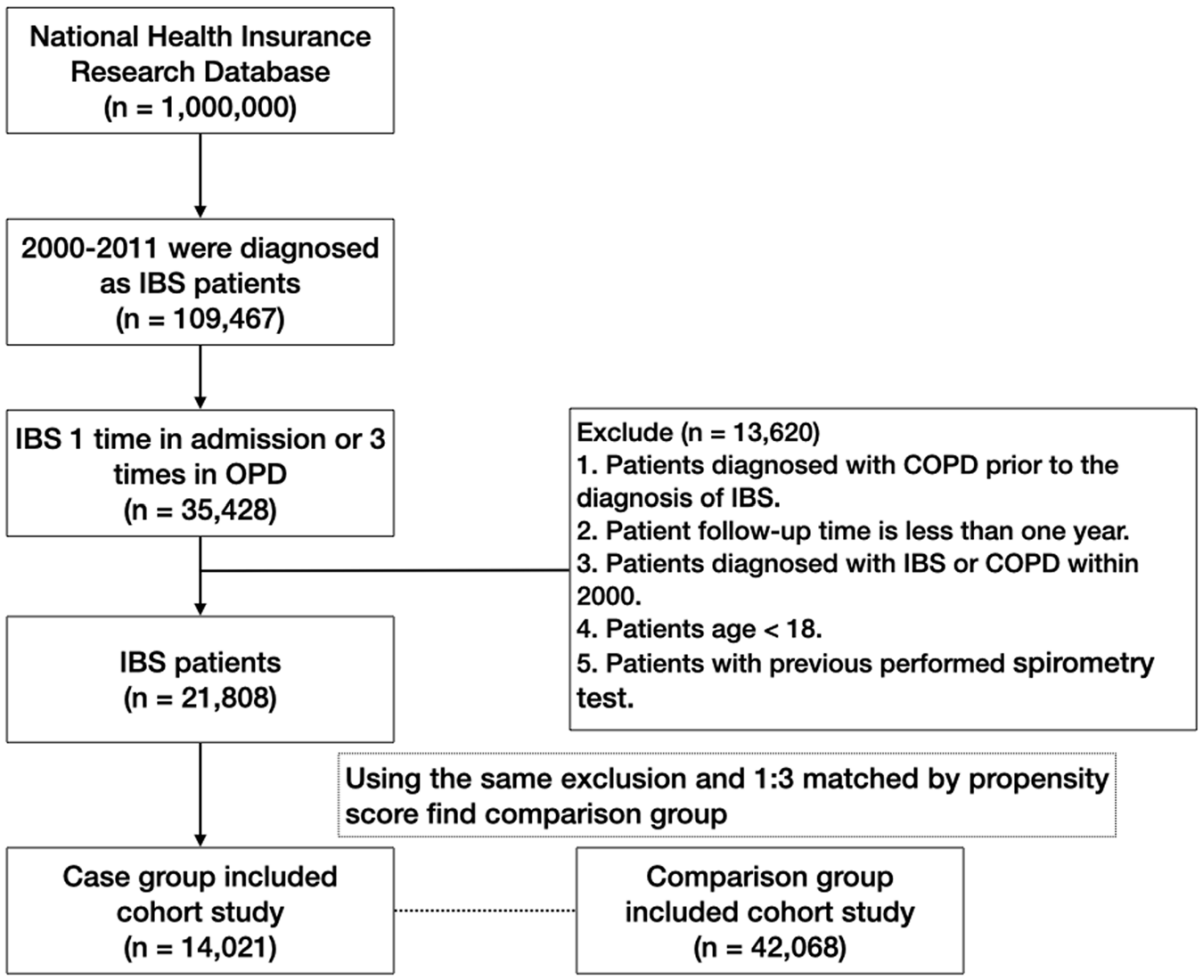
Flowchart of IBS (irritable bowel syndrome) and non-IBS enrollment from National Health Insurance Database (NHIRD) in Taiwan during 2000-2011.

Study population and variables. This is a retrospective cohort study conducted with the NHIRD. A flowchart illustrating subject selection for this study is shown in Fig. 3. Patients aged 18 years and older who were newly diagnosed with irritable bowel syndrome (IBS) (ICD-9-CM code 564.1), with follow-up of at least one year between January 1, 2000, and December 31, 2011, were enrolled and yielded 109,467 patients. Of these, we selected 35,428 patients with at least 3 OPD records or IBS diagnosis in admission. Only patients with a diagnosis of IBS were selected to ensure diagnostic validity. We excluded patients younger than 18 years of age, with follow-up of less than one year, with pre-existing COPD (ICD-9-CM code 491, 492, 496) before the diagnosis of IBS, with COPD within 1 year of IBS diagnosis, diagnosed with IBS or COPD within 2000, with previous performed spirometry test (code: 17003C, 17004B, 17006B, 17007B, 17019C) and those patients withdrawn from the NHIRD before diagnosis of IBS. These exclusion criteria yielded 13,620 patients for enrollment in the study. We then collected and analyzed demographic characteristics and claims data of both the IBS and non-IBS cohorts, matched by propensity score of 1:3. The case group and control group, consisting of 14,021 and 42,068 patients respectively, were included for further analysis. This study was approved by the Review Board and Ethics Committee of Taipei Medical University, Taiwan (TMU-JIRB No.: N201712044). All the datasets were decoded, so that the review board waived the requirement to sign informed consent from patients. All methods were carried out in accordance with relevant guidelines and regulations by ethics committee.

The demographic characteristics of the case and control cohorts including sex, age, residential area, salary, smoking-related diseases/conditions, common comorbidities, and drug intervention were taken into consideration as covariates to correct the analysis. The residential areas were divided into four main regions: northern Taiwan; central Taiwan; southern Taiwan; and eastern Taiwan. The study population was divided into six age groups: 18–29; 30–39; 40–49; 50–59; 60–69; and>69 years old. The monthly salary income was categorized into 3 groups: ≤NT$15,840, NT$15,841–25,000, ≥NT$25,001. Smoking status were defined as smoking-related diseases/conditions including personal history of tobacco use, tobacco use disorder, nonspecific abnormal results of pulmonary function studies, and other diseases of the lung (ICD 9: V15.82, 305.1, 794.2, 518) that are often diagnosed in the quit smoking clinic. Certain comorbid diseases were also recognized, including hypertension (ICD-9-CM code 401-405), coronary heart disease (ICD-9-CM code 410-414), ischemic stroke (ICD-9-CM code 433,434,436,437), hemorrhagic stroke (ICD-9-CM code 430-432), diabetes mellitus (ICD-9-CM code 250), renal insufficiency (ICD-9-CM code 585, 586, 588.8, 588.9), depression (ICD-9-CM code 296.2–296.36, 296.82, 300.4, 309.0, 309.1), asthma (ICD-9-CM code 493), Gastroesophageal reflux disease (GERD) (ICD-9-CM code 530.11, 530.81), peptic ulcer (ICD-9-CM code 531, 533), anxiety (ICD-9-CM code 293.84, 300.0, 300.00, 300.02, 300.09, 309.21), sleep apnea (ICD-9-CM code 780.51, 780.53, 780.57), and osteoporosis (ICD-9-CM code 733–733.09). The common use of COPD medications including steroids, anti-cholinergics, and beta-2 agonists were also recruited to test categorical variables.

### Statistical analysis

To reduce bias associated with confounding variables, we used propensity score-matched methods. To balance the measured covariate distribution in the cohorts, a propensity score for each patient was calculated by sex, age, area, urban, salary, smoking-related diseases/conditions, common comorbidities, and drug use. The propensity scores were calculated by using univariate and multivariate logistic regression to predict the probability of the occurrence of IBS. As such, the patients with IBS in line with 3 propensity score-matched subjects without IBS were comprised for further comparison.

The variables of the Cox regression model were used in this study. Categorical variables were demonstrated with counts and percentages, and those were further examined by Chi-square test to assess the association between the IBS and non-IBS cohorts. The mean age between the two groups was tested by two sample Student’s t-test. Univariate and multivariable Cox regression was used to assess the risk of COPD and estimated hazard ratios (HRs) with confidence intervals (95% CIs) between the two cohorts. In multivariate Cox regression, sex, age, salary, residential areas, smoking-related diseases/conditions, all comorbidities, and COPD medications were considered via adjustment. The Kaplan-Meier method to compare the cumulative incidence rate between IBS and non-IBS was applied for further management. The statistical analyses were performed using SAS software, version 9.4 (SAS Institute, Inc., Cary, NC, USA) and R software Version 3.4.3 (http://www.r-project.org), with level α = 0.05 to indicate statistical significance.

## Data Availability

The datasets used and/or analysed during the current study are available from the corresponding author on reasonable request.
